# Electronic, Optical, Thermoelectric and Elastic Properties of Rb_x_Cs_1−x_PbBr_3_ Perovskite

**DOI:** 10.3390/molecules28072880

**Published:** 2023-03-23

**Authors:** Elmustafa Ouaaka, Mustapha Aazza, Aziz Bouymajane, Francesco Cacciola

**Affiliations:** 1 Team of Renewable Energies, LP2MS Laboratory, Department of Physics, Faculty of Sciences, Moulay Ismail University, B.P 11201, Meknes 50070, Morocco; 2 Laboratory of Chemistry-Biology Applied to the Environment, Faculty of Sciences, Moulay Ismail University, B.P 11201, Meknes 50070, Morocco; 3 Team of Microbiology and Health, Laboratory of Chemistry-Biology Applied to the Environment, Faculty of Sciences, Moulay Ismail University, B.P 11201, Meknes 50070, Morocco; 4 Department of Biomedical, Dental, Morphological and Functional Imaging Sciences, University of Messina, 98125 Messina, Italy

**Keywords:** band structure, density functional theory, optical conductivity, electrical conductivity

## Abstract

Inorganic halide perovskites of the type AMX_3_, where A is an inorganic cation, M is a metal cation, and X is a halide anion, have attracted attention for optoelectronics applications due to their better optical and electronic properties, and stability, under a moist and elevated temperature environment. In this contribution, the electronic, optical, thermoelectric, and elastic properties of cesium lead bromide, CsPbBr_3_, and Rb-doped CsPbBr_3_, were evaluated using the density functional theory (DFT). The generalized gradient approximation (GGA) in the scheme of Perdew, Burke, and Ernzerhof (PBE) was employed for the exchange–correlation potential. The calculated value of the lattice parameter is in agreement with the available experimental and theoretical results. According to the electronic property results, as the doping content increases, so does the energy bandgap, which decreases after doping 0.75. These compounds undergo a direct band gap and present an energies gap values of about 1.70 eV (x = 0), 3.76 eV (x = 0.75), and 1.71 eV (x = 1). The optical properties, such as the real and imaginary parts of the dielectric function, the absorption coefficient, optical conductivity, refractive index, and extinction coefficient, were studied. The thermoelectric results show that after raising the temperature to 800 K, the thermal and electrical conductivities of the compound RbxCs_1−x_PbBr_3_ increases (x = 0, 0.25, 0.50 and 1). Rb_0.75_Cs_0.25_PbBr_3_ (x = 0.75), which has a large band gap, can work well for applications in the ultraviolet region of the spectrum, such as UV detectors, are potential candidates for solar cells; whereas, CsPbBr_3_ (x = 0) and RbPbBr_3_ (x = 1), have a narrow and direct band gap and outstanding absorption power in the visible ultraviolet energy range.

## 1. Introduction

Material scientists have discovered that energy collection from low-cost sources utilizing the most effective methods has gained significant importance. As a result, it is necessary to define the materials’ fundamental characteristics, in order to understand how they function in real-world working devices. Solar energy and unused heat can both be harvested with a great potential. Therefore, the goal of fundamental material research is to investigate innovative materials with optimum optical and thermoelectric properties. Generally, halide perovskite compounds with the general formula AMX_3_, where A is an organic or inorganic cation (such as ion cesium (Cs^+^), ion methyl ammonium (MA), or ion formamidinium (FA)), M is a metal cation (such as Pb^2+^ or Sn^2+^), and X is a halide anion (such as I^−^, Br^−^, and Cl^−^), have attracted much attention in the past few years due to their potential applications as solar-cell absorbers [1,2,3], light-emitting diodes [4,5], photodetectors [6,7,8,9,10,11], and lasers [12]. They possess numerous important technological applications due to their remarkable optical [13,14], electronic [15], and ferroelectric properties [16,17]. Organic–inorganic hybrid perovskite solar cells (PSC) are still adversely affected by poor stability, which reduces their practical applications, as exemplified by [18,19]. These hybrid perovskites degrade because of the considerable impact that moisture, heat, and UV light have on their organic phase.

One effective way to improve the stability of such perovskite requires the use of inorganic materials to replace labile organic components. Recently, all-inorganic halide perovskites of the type CsMX_3_ have attracted great attention for their usefulness in perovskite solar cell PSC stability improvement. Cesium-based inorganic perovskites have gained popularity due to their significantly increased thermal stability. Formamidinium- or methylammonium-based perovskite films degrade quickly above 200 °C, but the inorganic CsPbI_3_ and CsPbBr_3_ perovskite absorbers can maintain their original composition and crystal structure under high temperatures of up to 400 °C, effectively preventing the decomposition of organic groups and further enhancing the performance and stability of the device. In addition, compared to organic–inorganic hybrid perovskite materials, inorganic perovskite materials exhibit superior photoelectric properties, such as high carrier mobility and a long carrier lifetime. When formed from cold-pressed pellets of annealed CsSnI_3_ polycrystalline material, cesium-based inorganic perovskites have been shown to have a high hole mobility (520 cm^2^ V/s) and electron mobility (530 cm^2^ V/s), whereas CsPbBr_3_ halide single-crystals could achieve an electron lifetime of 2.5 s and an estimated electron mobility of 1000 cm^2^ V/s [20].

According to the current research, component engineering could change the perovskite crystal surface or integrate new ions into the crystalline structure to replace one of its substituents [21,22,23]. According to ab initio calculations of the electronic structure of perovskites, the outer shell orbitals of the M-site and X-site are significantly responsible for determining the energy levels (conduction band and valence band), depending on their electronegativity difference [24,25,26]. Therefore, element substitution on the A-, M- and/or X-sites can drastically change the band structure and the accompanying optical and electrical properties, such as the light absorption coefficient, bandgap, and charge carrier diffusion length.

Inorganic cations doping in perovskite materials have proven to be a successful tactic for achieving passivation faults, enhancing stability, and improving device performance [27,28,29]. Zhao et al. investigated the impact of A-site ion doping on perovskites [30]. The results demonstrated that alkali metal cations (K^+^ and Na^+^) can enhance perovskite film quality and perovskite solar cells photovoltaic performance. The poor carrier transport properties of inorganic perovskite materials severely restrict the development of the corresponding perovskite solar cell solar performance. Nam et al. partially substituted out Cs^+^ for K^+^, which reduced the PbX_6_ octahedron volume and increased phase stability [31].

There are several cesium lead halide CsPbX_3_ perovskites that have been created and can be utilized for perovskite solar cells, including CsPbBr_3_ and CsPbCl_3_ [32,33,34,35,36]. Additionally, all-inorganic perovskite solar cells, such as CsSnI_3_ [37] and CsPbI_3_ [38], have been used. Trots et al. [39] investigated the RbPbI_3_ and CsPbI_3_ compounds by the means of synchrotron powder diffraction experiments and Rietveld refinement technique. They have shown that both compounds crystallize in orthorhombic P_nma_ symmetry, revealing almost the same relative change of the lattice parameters upon heating, with an expansion isotropically close to 600 K. Moreover, they have observed that CsPbI_3_ undergoes first-order reversible phase transformation, whereas no transitions in RbPbI_3_ were detected. Zhao et al. [40] incorporated the Rb^+^ ions into the lattice of CsPbCl_3_ quantum dots by partially substituting the sites of Cs^+^ ions by the modified hot injection method. They observed that the high photoluminescence yields of CsPbCl_3_ were improved from 5.7% to 13% with Rb^+^ doping. It was observed that the emission and absorption peaks of CsPbCl_3_ quantum dots shifted to the shorter wavelength, and with the increase of Rb^+^ doping concentration, the lifetime of CsPbCl_3_ quantum dots was prolonged.

Due to the suppressed luminescence from the deep-level defects, CsPbX_3_ (X = Br or Cl) perovskites produced with a single crystalline nature have been demonstrated to be desirable for high-resolution detection at room temperature (RT) [41]. Further, it has been reported that the bulk-recrystallized CsPbBr_3_ emits bright green radiation at room temperature and provides a greater space for the free carriers, which lowers the recombination rates and, consequently, the poor quantum yield [42]. Fatty acids have been shown to inhibit the formation of CsPbBr_3_ nanocrystals, providing a novel technique to adjust the visible optical properties [43]. The obvious function of CsPbBr_3_ for optical devices is also covered in numerous additional experimental publications that are readily available [44,45]. Babu et al. [13] calculated the optical, electronic, structural and elastic properties of CsCaCl_3_ using the full potential linearized augmented plane wave method in the density functional theory. They found that this compound has an indirect energy band gap with a mixed ionic–covalent bonding, optically isotropic and structurally anisotropic property. The values of the band gaps found with different methods are 5.29, 5.35, 5.43, and 6.93 eV using LDA, GGA-PBE, GGA-WC, and mBJ pseudopotentials, respectively. Chang and Park [15] explored the electronic and structural properties of an inorganic perovskite, CsPbX_3_ (where X = Cl, Br and I), and the lead-halide-based inorganic–organic (CH_3_NH_3_)PbX_3_ perovskites, using the first-principles calculations within the local density approximation. They found that the lattice constants for the cubic structure of CsPbX_3_ were smaller than the corresponding values for (CH_3_NH_3_)PbX_3_; however, the electronic structures of both kinds of perovskites were found to be similar. Murtaza et al. [46] studied the optical, electronic and structural properties of cubic CsPbX_3_ (X = Cl, Br and I) using DFT calculations. They found that all of these compounds are direct, with a wide bandgap located at the R-symmetry point, which decreases from Cl to I. The refractive index, reflectivity and zero frequency limits of dielectric function increase with the decrease in bandgap (from Cl to Br to I), while the absorption coefficient and maximum optical conductivity decrease. Duong et al. [47] have demonstrated a novel multiplication method with methylammonium (MA), formamidinium (FA), Cesium (Cs) and Rb, to achieve high efficiency 1.73 eV bandgap perovskite cells, with negligible hysteresis. Mahmood et al. [48] investigated the thermoelectric, optical and mechanical properties of CsPbX_3_ (X = F, Cl, Br) using DFT calculations. They found that the thermal (*k*) and electrical (*σ*) conductivities increase with the increasing of temperature, and the ratio $\frac{k}{\sigma }$ remains at a minimum. When the mechanical and thermodynamic stabilities decrease from CsPbF_3_ to CsPbBr_3_, the structural stability increases.

All-inorganic lead APbI_3_ perovskites (with A = K, Li, Na or Cs cations), made via a self-organization process approach at room temperature, were experimentally explored by Dimesso et al. They discovered that the A cation size has a small impact on how these APbI_3_ perovskites’ bandgap energies change. Rb and K atoms have similar atomic radii to Cs, thus the correlation effect may be minimal [49]. The bandgap energy for CsPbBr_3_ with a cubic crystal structure was calculated by Qian et al., using the density functional theory (DFT) approach. They determined that this bandgap energy is 1.75 eV [50]. The anion electronegativity is a significant additional consequence of anion exchange. The calculations for CsPbX_3_ have also been done by Castelli et al., where X is changed for every halide group [51]. Their calculations revealed that the bandgap energy increased as the electronegativity of the anions increased. However, it appeared that the lattice constant, rather than the electronegativity, had a greater impact on the bandgap energy in the case of organometal perovskites. This explains why a perovskite with an formamidinium cation has a higher bandgap energy than one with a methyl ammonium cation. It is crucial to look into the role of the A cations and the X anions in the formation of the electronic structure of APbX_3_ perovskites, particularly the valence band and conduction band, as well as crystal binding properties, which are in charge of the processes of light absorption and photo generation of charge carriers.

In the cubic perovskite structure with space group $P_{m\bar{3}m}$, cesium–lead halides have been observed experimentally [52,53]. While the lattice constants of CsPbCl_3_ and CsPbBr_3_ were predicted using the ionic radii of the respective ions, the structural, electrical, thermodynamic, and optical aspects of these compounds were experimentally examined [39,54]. Using the first-principles pseudopotential method with a local density approximation and an empirical tight binding scheme, the structural and electrical characteristics of these compounds were also computed [15].

The aforementioned discussion makes it clear that there is only a limited amount of theoretical research on the optical, elastic, thermoelectric, and electronic properties of Rb-doped cesium lead bromide compounds. However, to our knowledge, no research has been reported on the optoelectronic and thermoelectric properties of this perovskite by density functional theory (DFT).

The aim of the present work was to investigate the electronic, optical, elastic and thermoelectric properties of CsPbBr_3_ (CPB) and Rb-doped CsPbBr_3_, using density functional theory and the Boltzmann Transport Equation (BTE) simulations. The differences between a pure CsPbBr_3_ and doped CPB (Rb_x_Cs_1−x_PbBr_3_), as well as the influence of Rb doping on these properties, are also discussed.

## 2. Results and Discussion

### 2.1. Structural Properties

According to earlier research, AMX_3_-type compounds display several phases at various temperatures, although at high temperatures, they all take on a cubic perovskite structure, where a three-dimensional framework of MX_6_ octahedrons with shared corners is provided.

The cubic structure phase of the perovskite CsPbBr_3_ compound has a space group $P_{m\bar{3}m}$ (221) and lattice parameter a = 5.605 Å.

**Figure 1:**
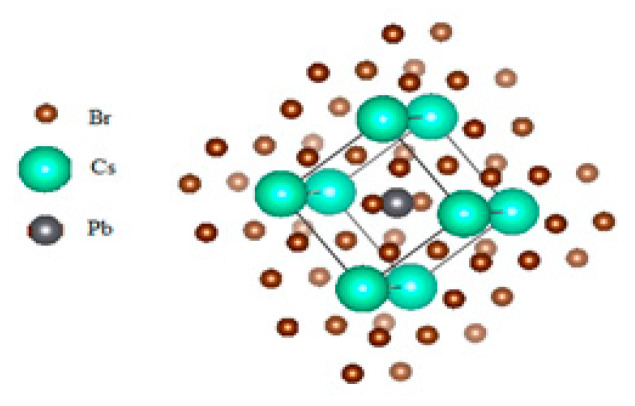
Crystal structure of CsPbBr_3_.

Figure 1 shows the crystal structure of CsPbBr_3_. The structure of the cubic CsPbBr_3_ compound was optimized. The optimization results for the lattice parameter are shown in Table 1 and are proved to be in reasonable agreement with experimental and theoretical values.

### 2.2. Elastic and Electronic Properties

For describing the mechanical properties of materials, the elastic constants are essential and basic. The elastic constants are significant factors that describe how macroscopic stress is responded to. In addition to defining how a material is deformed under stress and subsequently recovered and returned to its original shape after tension is removed, the elastic constants of solids also serve as a link between the mechanical and dynamical behavior of crystals. The elastic constants are significant material characteristics that can provide vital details about a material’s structural stability, the nature of its atomic bonds, and its anisotropic properties. There are three distinct elastic constants for a cubic system: B_11_, B_12_, and B_44_.

The state and behavior of the electrons in the material are completely described by a collection of characteristics and representations known as the electronic properties. Such a representation is, for instance, the electronic band structure, which characterizes the state of the electrons in terms of their energy, E, and momentum, k. The electric and optical characteristics, which define how a material reacts to electromagnetic radiation, are both closely related to the electronic properties. Examples of these are electrical conductivity and dielectric response.

**Figure 2:**
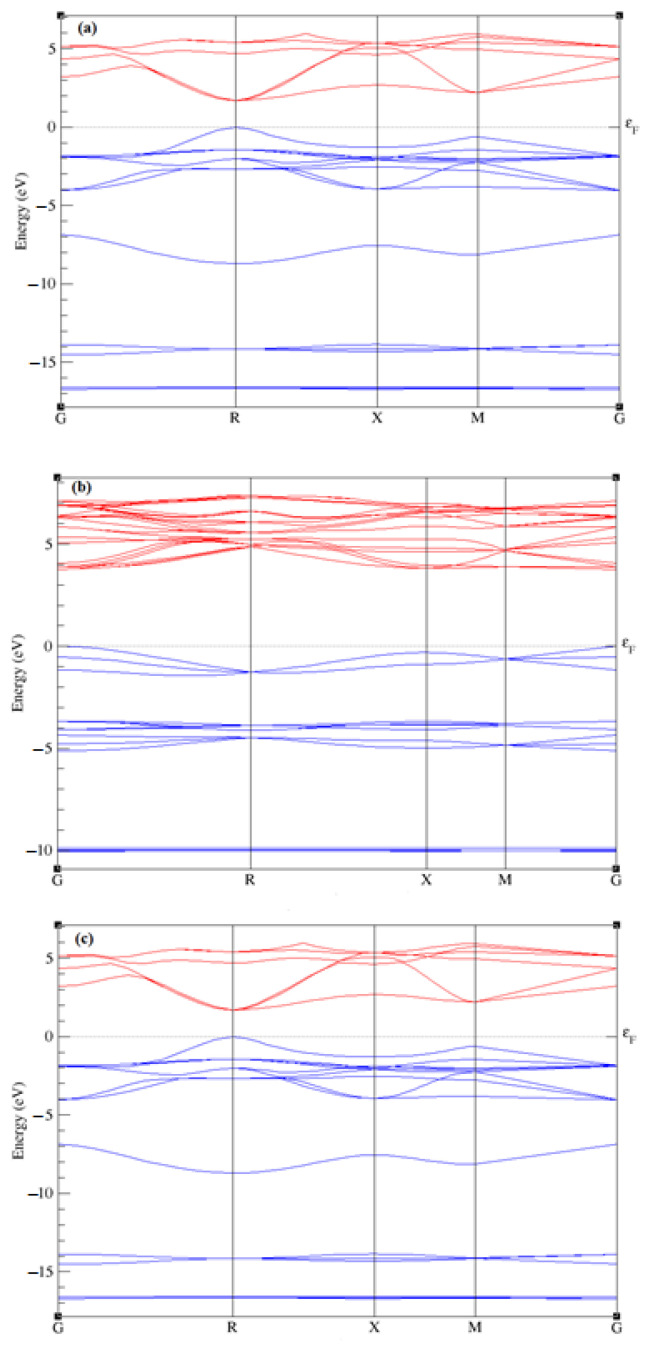
Band structures of CsPbBr_3_ and Rb_x_Cs_1−x_PbBr_3_ (x = 0 (**a**), 0.75 (**b**), 1 (**c**)). Valence bands (blue lines) and conduction bands (red lines).

**Figure 3:**
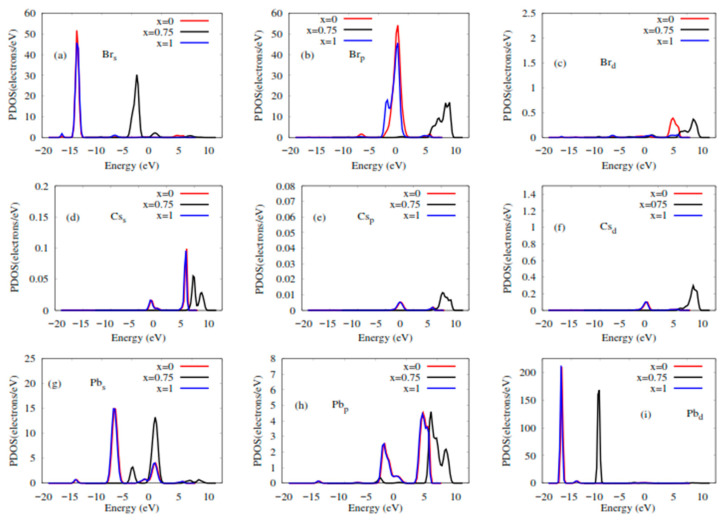
Partial density of states (PDOSs) of RbxCs_1−x_PbBr_3_ (x = 0, 0.75 and 1). (**a**) Br_s_ orbitals; (**b**) Br_p_ orbitals; (**c**) Br_d_ orbitals; (**d**) Cs_s_ orbitals; (**e**) Cs_p_ orbitals; (**f**) Cs_d_ orbitals; (**g**) Pb_s_ orbitals; (**h**) Pb_p_ orbitals; (**i**) Pb_d_ orbitals vs. energy.

The elastic and electronic properties of CsPbBr_3_, and Rb-doped CsPbBr_3_, including density of states and band structures, are calculated after the optimization of the lattice parameters. Table 2 lists the values of the elastic constants calculated via DFT calculations. Figure 2 shows the calculated electronic band structures of CsPbBr_3_ and Rb-doped CsPbBr_3_ along the higher symmetry directions G, R, X, and M. From the investigation results of CsPbBr_3_, Castelli et al. [51] reported that the bandgap energy for this perovskite is 1.63 eV, while Qian et al. [55] reported 1.75 eV. The present calculation results for CsPbBr_3_ (Figure 2a), which was 1.70 eV, gave a closer value to their computational results. Experimentally, Kulbak et al. [56] reported 2.32 eV regarding the bandgap energy of CsPbBr_3_, whereas Stoumpos et al. [45] reported 2.25 eV. On the other hand, the band structures of Rb_x_Cs_1−x_PbBr_3_ (x = 0.75 and 1) were calculated and shown in Figure 2b,c, respectively. It is clearly seen that these perovskites exhibited a direct band gap (Figure 2a–c), and achieved around 1.70, 3.76, and 1.71 eV, respectively, upon an increase in Rb content. Distinctions regarding the band gap energies are attributed to the atomic level and size. Furthermore, the gap energy observed in Figure 2c is lowered compared to the previous figures after an increase in the Rb-doping (x = 1), due to several mechanisms, such as the size of Rb. The results for CsPbBr_3_ (Figure 2a) are in good agreement with the experimental values [45,56,57]. The increase in the energy band gap, followed by a decrease as the doping content increases, is due to either octahedron tilting or a decrease in the overlap of the electron wave function, due to crystal structure contraction and distortion caused by the doping rubidium (Rb) atom [58,59]. To understand the electronic band gap nature, the densities of states (DOSs) of Rb_x_Cs_1−x_PbBr_3_ (x = 0, 0.75 and 1) were calculated and displayed in Figure 3. As can be appreciated, the valence bands in CsPbBr_3_ (x = 0) and RbPbBr3 (x = 1) are mostly composed of Brs, Brp, Pbs, Pbp, and Pbd orbitals, with a small contribution from Css, Csp, and Csd states. The conduction bands in both systems are mainly dominated by Pbp and Css orbitals, with small contributions of Brd and Brp. While in Rb_0.75_Cs_0.25_PbBr_3_ (x = 0.75), the valence band consists mainly of the orbital contributions Br-s, Pb-s, and Pb-d. The conduction band consists of the Pbp, Brp, and Brd orbitals, with small Css, Csp, and Csd states. It has been noted that the p orbitals of Pb and Br in the conduction band maximum have an effect on increasing the band gap in the 0.75 doping of Rb.

### 2.3. Optical Properties

Using solar cells and other optoelectronic devices, it is possible to directly convert the impinging photons into electricity. This capability has motivated scientists to look for materials with higher energy conversion efficiency. Direct inter-band transitions in direct band gap semiconductors are crucial, because indirect band gap semiconductors with intra-band transitions cause heating from phonons. This is because the optical properties of a typical semiconductor depend on the band gap, making transition or recombination rates crucial.

From the complex dielectric function, the optical properties of the halide perovskite materials CsPbBr_3_ and RbPbBr_3_ were theoretically studied. At a lower energy expression of complex, the dielectric function is:

(1) $$\varepsilon \left(\omega \right)=\varepsilon_{1}\left(\omega \right)+\varepsilon_{2}\left(\omega \right)$$

where $\varepsilon_{1}\left(\omega \right)$ and $\varepsilon_{2}\left(\omega \right)$ are the real and imaginary part of the dielectric function, respectively.

The real and imaginary part of the dielectric tensor can be estimated using the Kramer–Kronig relation:

(2) $$\varepsilon_{1}\left(\omega \right)=1+\frac{2}{\pi }*\int_{0}^{\infty }\frac{\varepsilon_{2}\left(\omega^{\prime }\right)\omega^{\prime }d\omega^{\prime }}{\left({\omega^{\prime }}^{2}-\omega^{2}\right)}$$

(3) $$\varepsilon_{2}\left(\omega \right)=\frac{8}{2\pi \omega^{2}}\sum {\left|P_{nn^{\prime }}\left(k\right)\right|}^{2}\frac{dS_{k}}{\nabla \omega_{nn^{\prime }}\left(k\right)}$$

(4) $$n\left(\omega \right)=\frac{1}{\sqrt{2}}{\left(\sqrt{\varepsilon_{1}{\left(\omega \right)}^{2}+\varepsilon_{2}{\left(\omega \right)}^{2}}+\varepsilon_{1}\left(\omega \right)\right)}^{\frac{1}{2}}$$

(5) $$k\left(\omega \right)=\frac{1}{\sqrt{2}}{\left(\sqrt{\varepsilon_{1}{\left(\omega \right)}^{2}+\varepsilon_{2}{\left(\omega \right)}^{2}}-\varepsilon_{1}\left(\omega \right)\right)}^{\frac{1}{2}}$$

The absorption coefficient *α* can be expressed as a function of the extinction coefficient *k*:

(6) $$\alpha =\frac{2\omega k}{c}$$

**Figure 4:**
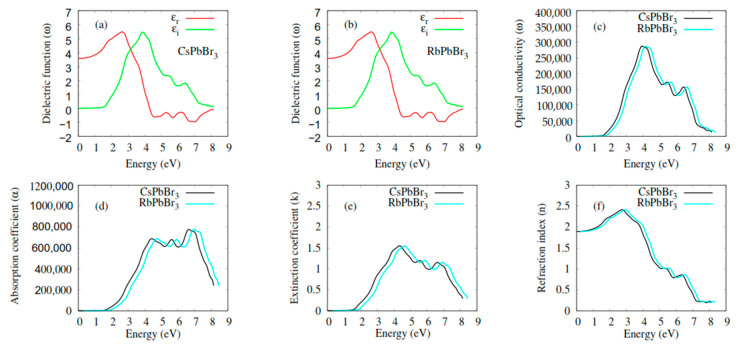
Optical properties of CsPbBr_3_ and RbPbBr_3_: (**a**,**b**) Dielectric function ($\varepsilon_{r}\left(\omega \right)$ and $\varepsilon_{i}\left(\omega \right)$); (**c**) optical conductivity σ(ω); (**d**) absorption coefficient α(ω); (**e**) extinction coefficient k(ω); (**f**) refaction index n(ω).

The imaginary part of the complex dielectric function, $\varepsilon_{2}\left(\omega \right)$, is related to the band structure of the material and describes its absorption behavior. From Figure 4a,b, the spectra of $\varepsilon_{2}\left(\omega \right)$ for CsPbBr_3_ and RbPbBr_3_ had similar features: the critical points (onset) in the spectra of $\varepsilon_{2}\left(\omega \right)$ were found at 1.66 eV for CsPbBr_3_ and RbPbBr_3_. These points are closely related to the band gap 1.70 eV for CsPbBr_3_ and RbPbBr_3_. Different characteristic peaks, beyond the critical points, could be identified by the density of states (Figure 3). The first peaks were due to the transition of electrons from Br_p_ states of the VB to the Pb_p_ states in the CB. The other peaks originated because of the electronic transition from Br_p_ states of VB to the unoccupied Cs_(s;d)_ and Rb_(s;d)_ states, and its mixed states with Pb_p_ states in CB. Interestingly, similar features were found in the spectra (Figure 4e) of the extinction coefficients, k(ω).

The real part of the complex dielectric function, $\varepsilon_{1}\left(\omega \right)$, is shown in Figure 4a,b. The most important quantity in the spectra is the zero-frequency limit $\varepsilon_{1}\left(0\right)$, which is the electronic part part of the static dielectric constant. The value $\varepsilon_{1}\left(0\right)$, for CsPbBr_3_ and RbPbBr_3_ was 3.5. The $\varepsilon_{1}\left(\omega \right)$, of CsPbBr_3_ and RbPbBr_3_ started to increase from the zero-frequency limit, reached its maximum value, then decreased, and in certain energy ranges, went below zero. The optical conductivity spectra, σ(ω) presented in Figure 4c, showed that the optical conductance started at around 1.52 and 1.58 eV for CsPbBr_3_ and RbPbBr_3_, respectively. Beyond these points, σ(ω) reached its maxima and then, again, decreased gradually. These compounds had a similar highest σ(ω). Similar features were observed regarding the absorption coefficients α(ω) (Figure 4d) in the range 0–6 eV, but the highest peaks were observed in the absorption range 6–9 eV of α(ω). Furthermore, the absorption range 2–8 eV showed the usefulness of CsPbBr_3_ and RbPbBr_3_ for various optical and optoelectronic devices working in this range. For an optical material to be used in optical devices, such photonic crystals, waveguides, solar cells, and detectors, it is crucial to understand the refractive index it has. The variation in the refractive indexes (*n*) for CsPbBr_3_ and RbPbBr_3_, as a function of incident photon energy, is shown in Figure 4f. The most important quantity in the spectra is the zero-frequency limit *n*(0), and its value is 2 for both CsPbBr_3_ and RbPbBr_3_. The n(ω) for these compounds increased gradually from the zero-frequency limit, reaching its maximal value, before decreasing. The theoretical analysis of CsPbBr_3_′s optical characteristics were equivalent to experimental analysis [63,64,65].

### 2.4. Thermoelectric Properties

The exponential growth of technology has led to enormous energy waste as a result of rising energy demands. Researchers have also been forced to create unique systems that can recycle waste heat into electrical energy due to the lower capacity of the available energy sources. One of the finest options is a thermoelectric generator, which can transform temperature gradients (phonons) directly into potential differences. The computed electrical conductivity measures the free carrier motion that results from temperature gradients that increase the carriers’ kinetic energy. A higher electrical conductivity is required to realize the commercial uses of thermoelectric devices, since it reduces the joule heating effect.

By comparing the ratio of heat efflux per area per unit time to the temperature gradient, it is possible to assess the flow of thermal energy. The two categories of thermal conductivity are electronic and phononic. Due to the importance of intra-band transitions in metals, as compared to semiconductors, lattice vibrations (phonons) have a greater influence in metals than they do in semiconductors. Additionally, whereas the phonon energy has little significance for direct bandgap semiconductors, it is significant for indirect bandgap semiconductors. In order to increase the efficiency of thermoelectric devices, the Wiedemann–Franz law specifies the minimal thermal-to-electrical conductivity ratio [66].

**Figure 5:**
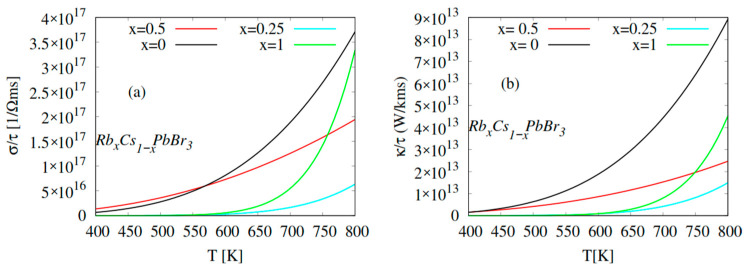
Thermoelectric properties of Rb_x_Cs_1−x_PbBr_3_ (x = 0, 0.25, 0.5, 1). (**a**) Electrical conductivity ($\frac{\sigma }{\tau }$) vs. temperature; (**b**) thermal conductivity ($\frac{k}{\tau }$) vs. temperature.

In the present work, to discuss the transport behavior of CsPbBr_3_ and Rb-doped CsPbBr_3_ compounds, the thermal $\frac{k}{\tau }$ and electrical $\frac{\sigma }{\tau }$ conductivities were calculated in the temperature range of 400–800 K, as displayed in Figure 5. It was observed that the electrical and thermal conductivities increased with increasing temperature until 800 K for pure and Rb-doped CPB. The decreasing slope of the electrical and thermal conductivity curves corresponding to Rb_0.25_Cs_0.75_PbBr_3_ and Rb_0.5_Cs_0.5_PbBr_3_ could be related to the increase of the band gap at T = 800 K, hence, the electrical conductivities at this temperature were 0.25 × 10^17^, 1.9 × 10^17^, 3.37 × 10^17^, and 3.75 × 10^17^ Ω^−1^ m^−1^s^−1^ for Rb_0.25_Cs_0.75_PbBr_3_, Rb_0.5_Cs_0.5_PbBr_3_, RbPbBr_3_, and CsPbBr_3_, respectively. At T = 800 K the thermal conductivities were 1.5 × 10^13^, 2.4 × 10^13^, 4.4 × 10^13^, and 9 × 10^13^ WK^−1^ m^−1^s^−1^ for Rb_0.25_Cs_0.75_PbBr_3_, Rb_0.5_Cs_0.5_PbBr_3_, RbPbBr_3_, and CsPbBr_3_, respectively. Our results are in good agreement with the experimental and theoretical studies reported in by [48,67,68,69], notably electrical conductivity. The increasing slope of the electrical conductivity curves from Rb_0.25_Cs_0.75_PbBr_3_ to RbPbBr_3_ is justified by the variation in the size of the atomic by effect of doping, which varies the free charge carriers.

## 3. Materials and Methods

New perovskite materials and their properties must be efficiently discovered using computational tools. Considering the wealthy amount of data, it is reasonable that this trend of employing computational methods will continue. Calculating the characteristics of materials is now possible without using experimental methods, because of the density functional theory (DFT). In physics and material science, DFT is a quantum mechanical modeling technique, that is employed to look into the electronic structure of many-body systems. In DFT, the exchange–correlation function, which is a mathematical approximation of the many-body effects of electron correlation, is used to treat electron correlation. In contrast, electron correlation is not included in HF, which reduces accuracy, but simplifies computation. As a result, it is probable that DFT is generally more accurate for many different calculations than HF, especially for systems with strong electron correlations [70]. DFT was realized in the 1980s by Pierre Hohenberg and Walter Kohn [71]. It is a commonly used computational method in material science for quickening the development of new materials and performing high-throughput simulations [72].

The electronic and optical properties of CsPbBr_3_ and Rb-doped CsPbBr_3_ perovskite were studied using DFT calculations, implemented in the ABINIT software package [73,74], with generalized gradient approximation (GGA) in the Perdew–Burke–Ernzerhof function, proposed in [75], using the plane wave pseudo-potential formalism, in order to obtain the response function calculations [76,77]. An energy cut-off of 45 Ha was used for the plane wave expansion, which are well converged. The Monkhorst Pack Mesh scheme [78] k-point grid sampling was set at 5 × 5 × 5, to perform the irreducible Brillouin zone integrations. We use a starting point for CsPbBr_3_ according to the reported data in the literature [79]. The thermoelectric properties were calculated using BoltzTraP code [80].

## 4. Conclusions

In this work, a systematic investigation of the electronic, optical, thermoelectric, and elastic properties of cesium lead bromide CsPbBr_3_ and Rb_x_Cs_1−x_PbBr_3_ (x = 0, 0.25, 0.50, 0.75, and 1) was carried out, using the density functional theory within the generalized gradient approximation and the Boltzmann transport equation simulations. The optical properties, such as dielectric function, optical conductivity, absorption coefficient, refractive index, and extinction coefficient, were studied in the energy range of 0–10 eV. The calculated band gap energy agrees well with the available theoretical and experimental values, and it increased then decreased as the Rb doping content increased. Our calculations revealed that Rb_0.75_Cs_0.25_PbBr_3_ is a wide band gap material, which indicates that it is a better candidate for high-frequency UV device applications.

CsPbBr_3_ (x = 0) and RbPbBr_3_ (x = 1), which have excellent absorption powers in the visible ultraviolet energy range and a short and direct band gap, could be used in solar cells.

## Figures and Tables

**Table 1:** Lattice parameter of CsPbBr_3_ compared to other calculations.

Present Work a (Å)	Other Works a (Å)	Experimental a (Å)
5.93	5.74 [15] 5.84 [29] 5.94 [60] 5.99 [58,60]	5.87 [60] 5.90 [57]

**Table 2:** Elastic properties of CsPbBr_3_ and gap energies of Rb_x_Cs_1−x_PbBr_3_ (x = 0, 0.75 and 1).

Elastic Constants (GPa)	Theoretical E_g_ (eV)	Experimental E_g_ (eV)
B_11_ = 163.30 B_12_ = 84.51 B_44_ = 10.93	1.70 (x = 0), 3.76 (x = 0.75) and 1.71 (x = 1): our work 1.769, 1.756: GGA-PBE [61] 1.148, 1.197: LDA-PZ [61]	2.383 [62] 2.25 [45], 2.36 [56], and 1.90 [57]

## Data Availability

Not applicable.

## Sample Availability

Samples of the compounds are not available from the authors.
